# Supplementary material for: Repositioning of Quinazolinedione-Based Compounds on Soluble Epoxide Hydrolase (sEH) through 3D Structure-Based Pharmacophore Model-Driven Investigation
Source: Molecules. 2022 Jun 16;27(12):3866. doi: 10.3390/molecules27123866 (PMC9228872; doi:10.3390/molecules27123866)
Supplement: Supplementary file 1 [file molecules-27-03866-s001.zip › molecules-1740714-supplementary.pdf]

Supplementary Materials

Repositioning of Quinazolinedione-Based Compounds on Soluble Epoxide Hydrolase (sEH) through 3D Structure-Based Pharmacophore Model-Driven Investigation

Erica Gazzillo [1,†], Stefania Terracciano [1,†], Dafne Ruggiero [1], Marianna Potenza [2], Maria Giovanna Chini [3], Gianluigi Lauro [1], Katrin Fischer [4], Robert Klaus Hofstetter [4], Assunta Giordano [5], Oliver Werz [4], Ines Bruno [1,*] and Giuseppe Bifulco [1,*]

1 Department of Pharmacy, University of Salerno, via Giovanni Paolo II 132, 84084 Fisciano, SA, Italy; egazzillo@unisa.it (E.G.); sterracciano@unisa.it (S.T.); druggiero@unisa.it (D.R.); glauro@unisa.it (G.L.); asgiordano@unisa.icb.cnr.it (A.G.)
2 The FIRC Institute of Molecular Oncology, via Adamello 16, 20139 Milan, MI, Italy; marianna.potenza@ifom.eu
3 Department of Biosciences and Territory, University of Molise, C.da Fonte Lappone, 86090 Pesche, IS, Italy; mariagiovanna.chini@unimol.it
4 Department of Pharmaceutical/Medicinal Chemistry, Institute of Pharmacy, Friedrich-Schiller-University, Jena, Philosophenweg 14, 07743 Jena, Germany; katrin.fischer.1@uni-jena.de (K.F.); robert.klaus.hofstetter@uni-jena.de (R.K.H.); oliver.werz@uni-jena.de (O.W.)
5 Institute of Biomolecular Chemistry (ICB), Consiglio Nazionale delle Ricerche (CNR), via Campi Flegrei 34, 80078 Pozzuoli, NA, Italy
* Correspondence: brunoin@unisa.it (I.B.); bifulco@unisa.it (G.B.); Tel.: +39-089-969743 (I.B.); +39-089-969741 (G.B.)
† These authors contributed equally to this work.

Table of contents

Additional computational details S3

Figure S1. ....S4

Figure S2. ....S5

Figure S3. ....S5

Table S1. ....S6

References..S10

Additional computational details

Molecular docking experiments on Hsp90

The X-ray structure of human Hsp90 is not available in the Protein Data Bank (PDB). However, the structure-based sequence alignment between the yeast Hsp82 (PDB code: 2CG9)[1] and the human Hsp90α reported in 2011[2] highlighted about 60% identity in 438 residues and the C-terminal domains of the two chaperones feature a related root-mean square deviation (RMSD) = 0.91 Å for 219 Cα atoms[2]. For this reason, the Hsp82 X-ray structure was chosen, and the region including Arg670 of Hsp82, which corresponds to Lys656 of Hsp90 and belonging to the related binding site, was used for molecular docking experiments[3-9]. Specifically, for the selection of the most promising compounds, specific interactions at the interface of the two Hsp90 A and B chains were chosen as selection filters, namely hydrogen bonds with leucines (Leu671, Leu674, Leu676), and halogen interactions with Thr638$_{ChainB}$ and Asp641$_{ChainA}$, previously identified as fundamental interactions for the design of novel C-terminal Hsp90 inhibitors[10].

The selected crystal structure of the protein (PDB code: 2CG9)[1], complexed with the co-chaperone Sba1 in the active closed conformation, was deposited in 2006 and is characterized by a resolution of 3.1 Å[1]. The 3D protein model was prepared using the Schrödinger Protein Preparation Wizard[11,12]: hydrogens and cap termini were added, bond orders were assigned. The receptor grid used for docking calculation was characterized from inner and outer box dimensions of 10 × 10 × 10 and 30 × 30 × 30, respectively. The grid center coordinates were −61.32 × −53.34 × 35.98. Subsequently, the generated combinatorial library was submitted to the virtual screening workflow (VSW) using Glide software[13-16]. Specifically, VSW is performed at three levels of precision, HTVS, SP, and XP. For HTVS and SP, the first 60% of ranked compounds by docking score were saved for the next phases (SP and XP, respectively), accounting one docking pose for each compound; for the XP level, the 80% of top ranked poses were saved, accounting 10 poses as maximum number of solutions for each compound. Finally, the poses featuring a docking score higher than −2.0 kcal/mol from the best found value were visual inspected, and the most promising compounds were selected for the synthesis step.

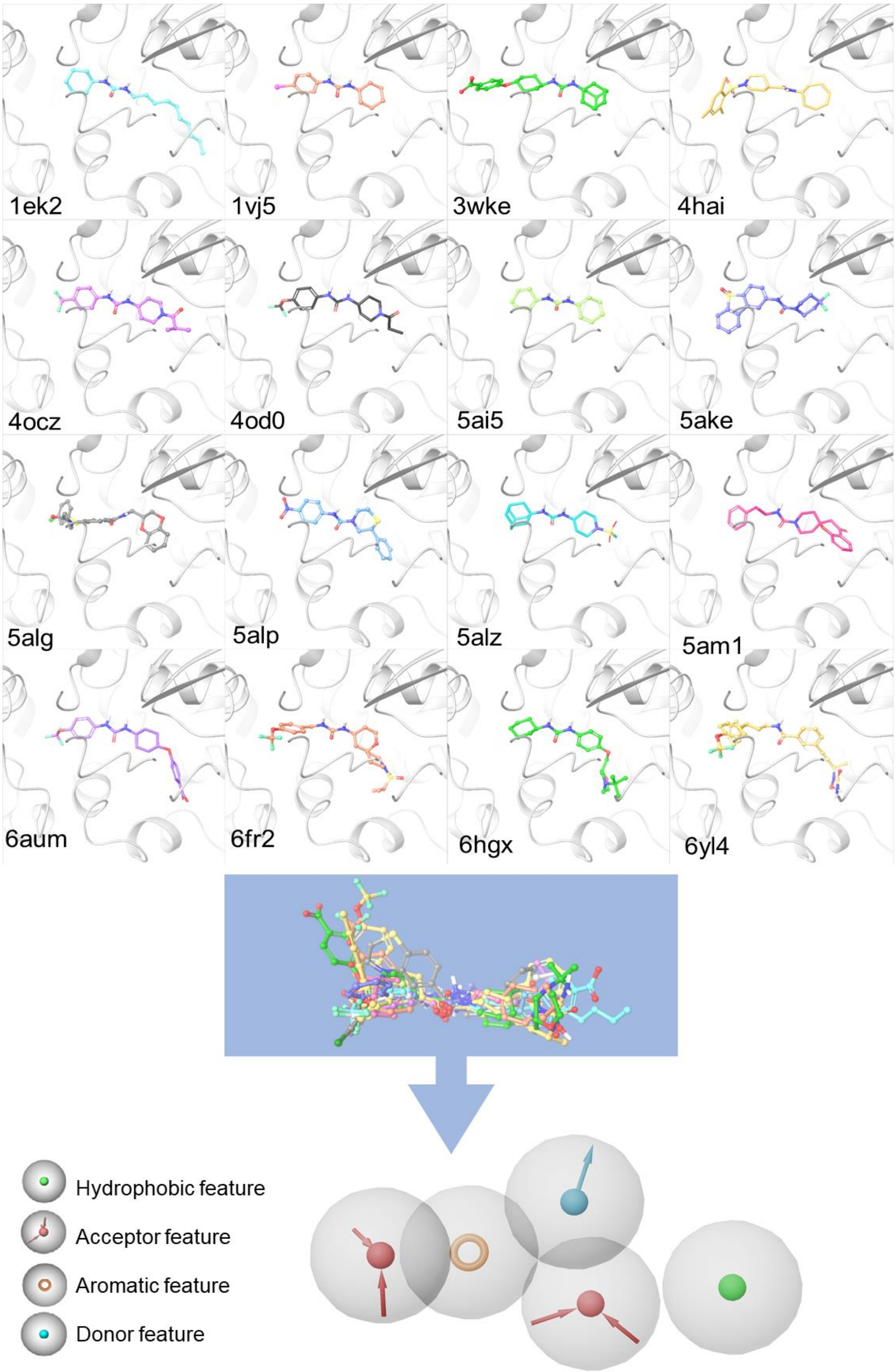

Figure S1. From top to bottom: 1) binding mode representation of 16 selected binders; 2) superimposed ligands; 3) pharm-sEH featuring the legend of the pharmacophoric features.

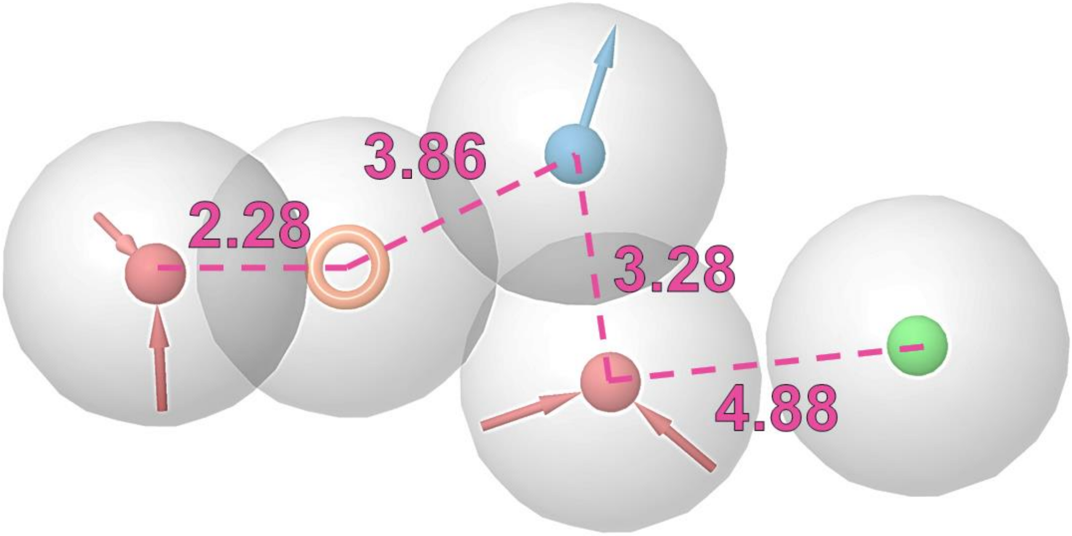

Figure S2. Distances (in Angstroms) between the key features of “pharm-sEH".

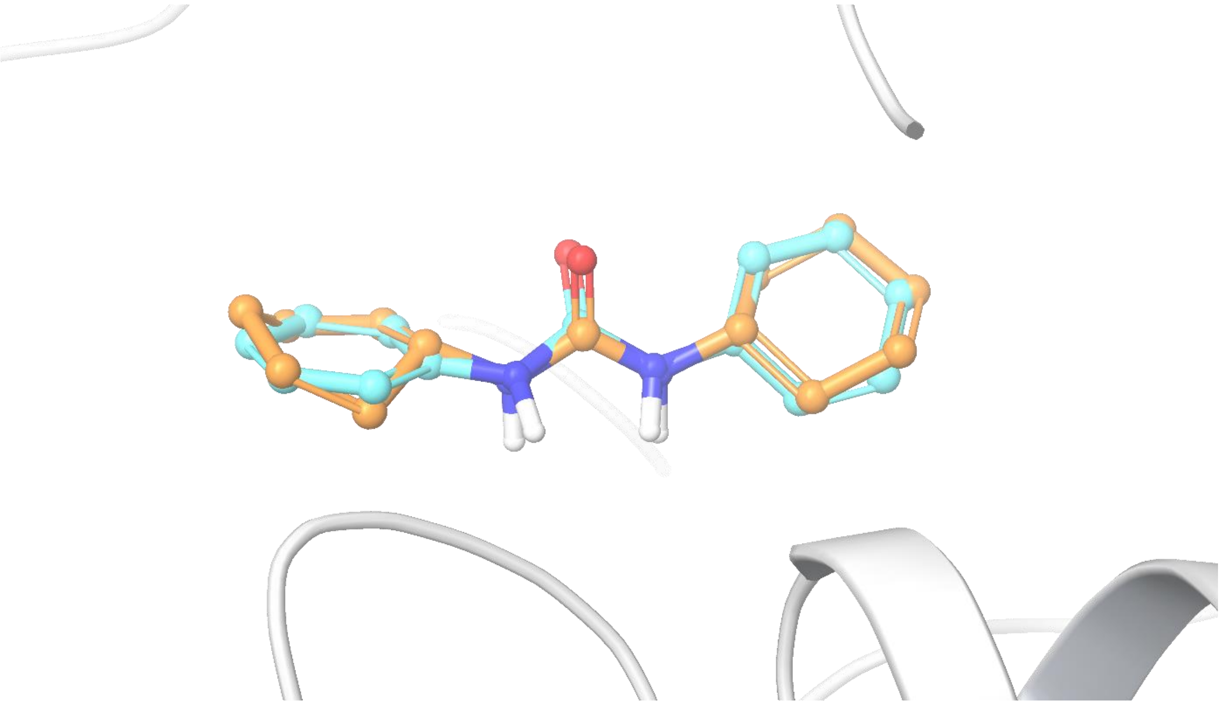

Figure S3. Superposition of co-crystallized BSU (PDB code: 5AI5) and redocked BSU for the validation of docking protocol.

Table S1. SMILES of the compounds belonging to the investigated in-house library.

Compound	SMILES
**3**	O1CCOc(c12)ccc(c2)NC(=O)CCCCn(c3=O)c(=O)[nH]c(c34)cccc4
**4**	O1CCOc(c12)ccc(c2)NC(=O)CCCCCn(c3=O)c(=O)[nH]c(c34)cccc4
**5**	c1ncc(Br)cc1NC(=O)CCCCn(c2=O)c(=O)[nH]c(c23)cccc3
**6**	c1ncc(Br)cc1NC(=O)CCCCCCn(c2=O)c(=O)[nH]c(c23)cccc3
**7**	c1nc(I)ccc1NC(=O)CCCCCn(c2=O)c(=O)[nH]c(c23)cccc3
**8**	n1csc(c12)cc(cc2)NC(=O)CCCCCn(c3=O)c(=O)[nH]c(c34)cccc4
**9**	CC(C)Oc(cc1)ccc1-c(cc2)ccc2-c3nc(N)nc(c34)[nH]cn4
**10**	CCOC(=O)C1=C(C)N(CCC([O-])=O)C(=O)N[C@@H]1c2ccc([N+]([O-])=O)cc2
**11**	CCOC(=O)C1=C(C)N(CCC([O-])=O)C(=O)N[C@@H]1c2ccccn2
**12**	CCOC(=O)C1=C(C)N(CCC([O-])=O)C(=O)N[C@@H]1c(c2=O)coc(c23)ccc(Br)c3
**13**	CCOC(=O)C1=C(C)N(CCC([O-])=O)C(=O)N[C@@H]1c(cc2C([O-])=O)ccc2
**14**	CCOC(=O)C1=C(C)N(CCC([O-])=O)C(=O)N[C@@H]1c(n2)cccc2-c(ccc3)cc3C(=O)OC
**15**	CCOC(=O)C1=C(C)N(CCC([O-])=O)C(=O)N[C@H]1c2ccc([N+]([O-])=O)cc2
**16**	CCOC(=O)C1=C(C)N(CCC([O-])=O)C(=O)N[C@H]1c2ccccn2
**17**	CCOC(=O)C1=C(C)N(CCC([O-])=O)C(=O)N[C@H]1c(c2=O)coc(c23)ccc(Br)c3
**18**	CCOC(=O)C1=C(C)N(CCC([O-])=O)C(=O)N[C@H]1c(cc2C([O-])=O)ccc2
**19**	CCOC(=O)C1=C(C)N(CCC([O-])=O)C(=O)N[C@H]1c(n2)cccc2-c(ccc3)cc3C(=O)OC
**20**	CCOC(=O)C1=C(C)N(c2ccccc2)C(=O)N[C@@H]1c3c(Br)cc(c(c3)O)OC
**21**	CCOC(=O)C1=C(C)N(c2ccccc2)C(=O)N[C@@H]1c3c(Br)ccc(c3)O
**22**	CCOC(=O)C1=C(C)N(c2ccccc2)C(=O)N[C@@H]1c3c(O)ccc(c3)OC
**23**	CCOC(=O)C1=C(C)N(c2ccccc2)C(=O)N[C@@H]1c3c([N+]([O-])=O)ccc([O-])c3
**24**	CCOC(=O)C1=C(C)N(c2ccccc2)C(=O)N[C@@H]1c3c([N+]([O-])=O)cccc3
**25**	CCOC(=O)C1=C(C)N(c2ccccc2)C(=O)N[C@@H]1c3cc(Br)ccc3
**26**	CCOC(=O)C1=C(C)N(c2ccccc2)C(=O)N[C@@H]1c3cc([N+]([O-])=O)ccc3
**27**	CCOC(=O)C1=C(C)N(c2ccccc2)C(=O)N[C@@H]1c3ccc(Br)cc3
**28**	CCOC(=O)C1=C(C)N(c2ccccc2)C(=O)N[C@@H]1c3ccc(Cl)cc3
**29**	CCOC(=O)C1=C(C)N(c2ccccc2)C(=O)N[C@@H]1c3ccc(O)cc3
**30**	CCOC(=O)C1=C(C)N(c2ccccc2)C(=O)N[C@@H]1c3ccc([N+]([O-])=O)cc3
**31**	CCOC(=O)C1=C(C)N(c2ccccc2)C(=O)N[C@@H]1c3ccc(cc3)OC
**32**	CCOC(=O)C1=C(C)N(c2ccccc2)C(=O)N[C@@H]1c(c3C(F)(F)F)cccc3
**33**	CCOC(=O)C1=C(C)N(c2ccccc2)C(=O)N[C@@H]1c(c(Br)cc3)cc3C(F)(F)F
**34**	CCOC(=O)C1=C(C)N(c2ccccc2)C(=O)N[C@@H]1c(c(Cl)cc3)cc3C(F)(F)F
**35**	CCOC(=O)C1=C(C)N(c2ccccc2)C(=O)N[C@@H]1c(cc3)ccc3C
**36**	CCOC(=O)C1=C(C)N(c2ccccc2)C(=O)N[C@H]1c3c(Br)cc(c(c3)O)OC
**37**	CCOC(=O)C1=C(C)N(c2ccccc2)C(=O)N[C@H]1c3c(Br)ccc(c3)O
**38**	CCOC(=O)C1=C(C)N(c2ccccc2)C(=O)N[C@H]1c3c(O)ccc(c3)OC
**39**	CCOC(=O)C1=C(C)N(c2ccccc2)C(=O)N[C@H]1c3c([N+]([O-])=O)ccc([O-])c3
**40**	CCOC(=O)C1=C(C)N(c2ccccc2)C(=O)N[C@H]1c3c([N+]([O-])=O)cccc3
**41**	CCOC(=O)C1=C(C)N(c2ccccc2)C(=O)N[C@H]1c3cc(Br)ccc3
**42**	CCOC(=O)C1=C(C)N(c2ccccc2)C(=O)N[C@H]1c3cc([N+]([O-])=O)ccc3
**43**	CCOC(=O)C1=C(C)N(c2ccccc2)C(=O)N[C@H]1c3ccc(Br)cc3
**44**	CCOC(=O)C1=C(C)N(c2ccccc2)C(=O)N[C@H]1c3ccc(Cl)cc3
**45**	CCOC(=O)C1=C(C)N(c2ccccc2)C(=O)N[C@H]1c3ccc(O)cc3
**46**	CCOC(=O)C1=C(C)N(c2ccccc2)C(=O)N[C@H]1c3ccc([N+]([O-])=O)cc3
**47**	CCOC(=O)C1=C(C)N(c2ccccc2)C(=O)N[C@H]1c3ccc(cc3)OC
**48**	CCOC(=O)C1=C(C)N(c2ccccc2)C(=O)N[C@H]1c(c3C(F)(F)F)cccc3
**49**	CCOC(=O)C1=C(C)N(c2ccccc2)C(=O)N[C@H]1c(c(Br)cc3)cc3C(F)(F)F
**50**	CCOC(=O)C1=C(C)N(c2ccccc2)C(=O)N[C@H]1c(c(Cl)cc3)cc3C(F)(F)F

**51**	CCOC(=O)C1=C(C)N(c2ccccc2)C(=O)N[C@H]1c(cc3)ccc3C
**52**	CCOC(=O)C1=C(C)NC(=S)N[C@@H]1c2cc(ccc2)OCC
**53**	CCOC(=O)C1=C(C)NC(=S)N[C@H]1c2cc(ccc2)OCC
**54**	CCOC(=O)CN(C(=O)S1)C(=O)\C1=C\c2c([O-])ccc(c2)[N+]([O-])=O
**55**	CCOC(=O)CN(C(=O)S1)C(=O)\C1=C\c2cc(O)c(O)cc2
**56**	CCOC(=O)CN(C(=O)S1)C(=O)\C1=C\c2cc(OCc3ccccc3)c(cc2)OCc4ccccc4
**57**	CCOC(=O)CN(C(=O)S1)C(=O)\C1=C\c2cc([O-])c([N+]([O-])=O)cc2
**58**	CCOc(ccc1)c([O-])c1/C=C2/C(C(C)C)=NN(C2=O)c3cnccn3
**59**	CCOc(ccc1)c([O-])c1/C=C2/C(CC)=NN(C2=O)c3c(F)cc(F)cc3
**60**	CCOc(ccc1)c([O-])c1/C=C2/C(CC)=NN(C2=O)c3cnccn3
**61**	COc1c(OC)cc(cc1OC)-c2nc(N)nc(c23)[nH]cn3
**62**	COc(c1)cc(OC)cc1COc(ccc2)cc2-c3nc(N)nc(c34)[nH]cn4
**63**	COc(c(c1)O)cc(Br)c1/C=C2/C(C)=NN(C2=O)c(cc3)ccc3[N+]([O-])=O
**64**	COc(cc1)ccc1/C=C/C(N=C(C)\C2=C(\[O-])OCC)=[NH+][C@@H]2c3cc(ccc3)OCC
**65**	COc(cc1)ccc1/C=C/C(N=C(C)\C2=C(\[O-])OCC)=[NH+][C@H]2c3cc(ccc3)OCC
**66**	COc(cc1)ccc1C(=O)NC(S2)=N[C@@H](c3ccc(F)cc3)C=C2c4ccccc4
**67**	COc(cc1)ccc1C(=O)NC(S2)=N[C@@H](c3ccccc3)C=C2c4ccccc4
**68**	COc(cc1)ccc1C(=O)NC(S2)=N[C@H](c3ccc(F)cc3)C=C2c4ccccc4
**69**	COc(cc1)ccc1C(=O)NC(S2)=N[C@H](c3ccccc3)C=C2c4ccccc4
**70**	FC(F)(F)Oc1ccc(cc1)OCc2cc(ccc2)-c3nc(N)nc(c34)[nH]cn4
**71**	FC(F)(F)c1cc(c(Cl)cc1)/C=C2/C(C)=NN(C2=O)c(cc3)ccc3[N+]([O-])=O
**72**	FC(F)(F)c1cc(c(Cl)cc1)OCc2cc(ccc2)-c3nc(N)nc(c34)[nH]cn4
**73**	FC(F)(F)c1cc(c(Cl)cc1)OCc2ccc(cc2)-c3nc(N)nc(c34)[nH]cn4
**74**	FC(F)(F)c1cc(ccc1)-c(o2)ccc2/C=C3/C(CC)=NN(C3=O)c(cc4C(F)(F)F)ccc4
**75**	FC(F)(F)c1cc(ccc1)CCn(nn2)cc2-c3cc(O)ccc3
**76**	FC(F)(F)c1cc(ccc1)CCn(nn2)cc2-c3ccccn3
**77**	FC(F)(F)c1cc(ccc1)COc(ccc2)cc2-c3nc(N)nc(c34)[nH]cn4
**78**	FC(F)(F)c1cc(ccc1)N(C2=O)N=C(C(C)C)\C2=C\c3cc([O-])c([N+]([O-])=O)cc3
**79**	FC(F)(F)c1cc(ccc1)N(C2=O)N=C(CC)\C2=C\c3c([O-])c(ccc3)OCC
**80**	FC(F)(F)c1cc(ccc1)N(C2=O)N=C(CC)\C2=C\c3c([O-])ccc(c3)[N+]([O-])=O
**81**	FC(F)(F)c1cc(ccc1)NC(=O)CN(C(=O)S2)C(=O)\C2=C\c3c([O-])cc(OC)cc3OC
**82**	FC(F)(F)c1ccc(cc1)/C=C/C(N=C(C)/C2=C(\[O-<br>])OCC)=[NH+][C@@H]2c3cc(ccc3)OCC
**83**	FC(F)(F)c1ccc(cc1)/C=C/C(N=C(C)/C2=C(\[O-])OCC)=[NH+][C@H]2c3cc(ccc3)OCC
**84**	N#Cc1cc(ccc1)-c(n2)cccc2/C=C3/C(C)=NN(C3=O)c(cc4)ccc4[N+]([O-])=O
**85**	N#Cc1ccc(cc1)N(C2=O)N=C(C)\C2=C\c3c([O-])ccc(c3)[N+]([O-])=O
**86**	O1COc(c12)ccc(c2)C(=O)OCc3c(C)n(nn3)-c4nccs4
**87**	O1COc(c12)ccc(c2)C(=O)OCc3cn(nn3)-c(cc4)cc(c45)scn5
**88**	O1COc(c12)ccc(c2)C(=O)OCc3cn(nn3)Cc4nc(no4)-c5cccnc5
**89**	O1COc(c12)ccc(c2)C(=O)OCc3cn(nn3)Cc4nnc(o4)-c5cccnc5
**90**	[O-]C(=O)c1cc([N+]([O-])=O)c(cc1)Cn(nn2)cc2-c3cc(O)ccc3
**91**	[O-]C(=O)c1cc([N+]([O-])=O)c(cc1)Cn(nn2)cc2-c3ccccn3
**92**	[O-]C(=O)c1cc(ccc1)/C=C2/C(C)=NN(C2=O)c(cc3)ccc3[N+]([O-])=O
**93**	[O-]C(=O)c1cc(ccc1)[C@@H]2NC(=O)N(C(C)=C2C(=O)OCC)c3ccccc3
**94**	[O-]C(=O)c1cc(ccc1)[C@H]2NC(=O)N(C(C)=C2C(=O)OCC)c3ccccc3
**95**	[O-][N+](=O)c1c(Cl)ccc(c1)NC(=O)CN(C(=O)S2)C(=O)\C2=C\c(n3)ccc(c34)cccc4[O-]
**96**	[O-][N+](=O)c(c1)ccc([O-])c1/C=C2/C(C)=NN(C2=O)c3c(F)cc(Br)cc3
**97**	[O-][N+](=O)c(c1)ccc([O-])c1/C=C2/C(C)=NN(C2=O)c(cc3[N+]([O-])=O)ccc3
**98**	[O-][N+](=O)c(c1)ccc([O-])c1/C=C2/C(CC)=NN(C2=O)c3c(F)cc(F)cc3
**99**	[O-][N+](=O)c(c1)ccc([O-])c1/C=C2/C(CC)=NN(C2=O)c3cnccn3
**100**	c1c(O)ccc(Br)c1/C=C2/C(C)=NN(C2=O)c(cc3)ccc3[N+]([O-])=O
**101**	c1cc(C)cc(c1C(C)C)OCc2ccc(cc2)-c3nc(N)nc(c34)[nH]cn4
**102**	c1cc(C)ccc1/C=C/C(N=C(C)/C2=C(\[O-])OCC)=[NH+][C@@H]2c3cc(ccc3)OCC

**103**	c1cc(C)ccc1/C=C/C(N=C(C)/C2=C(\[O-])OCC)=[NH+][C@H]2c3cc(ccc3)OCC
**104**	c1cc(C)ccc1C(=O)NC(S2)=N[C@@H](c3ccc(F)cc3)C=C2c4ccccc4
**105**	c1cc(C)ccc1C(=O)NC(S2)=N[C@@H](c3ccccc3)C=C2c4ccccc4
**106**	c1cc(C)ccc1C(=O)NC(S2)=N[C@H](c3ccc(F)cc3)C=C2c4ccccc4
**107**	c1cc(C)ccc1C(=O)NC(S2)=N[C@H](c3ccccc3)C=C2c4ccccc4
**108**	c1cc(Cl)ccc1/C=C2/C(C)=NN(C2=O)c(cc3)ccc3[N+]([O-])=O
**109**	c1cc(Cl)ccc1/C=C/C(N=C(C)/C2=C(\[O-])OCC)=[NH+][C@@H]2c3cc(ccc3)OCC
**110**	c1cc(Cl)ccc1/C=C/C(N=C(C)/C2=C(\[O-])OCC)=[NH+][C@H]2c3cc(ccc3)OCC
**111**	c1cc(Cl)ccc1C(=O)NC(S2)=N[C@@H](c3ccc(F)cc3)C=C2c4ccccc4
**112**	c1cc(Cl)ccc1C(=O)NC(S2)=N[C@@H](c3ccccc3)C=C2c4ccccc4
**113**	c1cc(Cl)ccc1C(=O)NC(S2)=N[C@H](c3ccc(F)cc3)C=C2c4ccccc4
**114**	c1cc(Cl)ccc1C(=O)NC(S2)=N[C@H](c3ccccc3)C=C2c4ccccc4
**115**	c1cc(F)ccc1/C=C/C(N=C(C)/C2=C(\[O-])OCC)=[NH+][C@@H]2c3cc(ccc3)OCC
**116**	c1cc(F)ccc1/C=C/C(N=C(C)/C2=C(\[O-])OCC)=[NH+][C@@H]2c3ccc(cc3)OCC=C
**117**	c1cc(F)ccc1/C=C/C(N=C(C)/C2=C(\[O-])OCC)=[NH+][C@H]2c3cc(ccc3)OCC
**118**	c1cc(F)ccc1/C=C/C(N=C(C)/C2=C(\[O-])OCC)=[NH+][C@H]2c3ccc(cc3)OCC=C
**119**	c1cc(F)ccc1/C=C/C(NC(C)=C2C(=O)OCC)=N[C@@H]2c3c(Br)ccc(c3)O
**120**	c1cc(F)ccc1/C=C/C(NC(C)=C2C(=O)OCC)=N[C@H]2c3c(Br)ccc(c3)O
**121**	c1cc(F)ccc1C(=O)NC(S2)=N[C@@H](c3ccc(F)cc3)C=C2c4ccccc4
**122**	c1cc(F)ccc1C(=O)NC(S2)=N[C@@H](c3ccccc3)C=C2c4ccccc4
**123**	c1cc(F)ccc1C(=O)NC(S2)=N[C@H](c3ccc(F)cc3)C=C2c4ccccc4
**124**	c1cc(F)ccc1C(=O)NC(S2)=N[C@H](c3ccccc3)C=C2c4ccccc4
**125**	c1cc(F)ccc1COc(ccc2)cc2-c3nc(N)nc(c34)[nH]cn4
**126**	c1cc(O)c(O)cc1/C=C2/C(=O)N(C(=O)S2)CCc3c[nH]c(c34)cccc4
**127**	c1cc(O)c(O)cc1/C=C2/C(=O)N(C(=O)S2)Cc3nnc(o3)-c4ccccn4
**128**	c1cc(O)cc(O)c1C(=O)OCc2c(C)n(nn2)-c3nccs3
**129**	c1cc([N+]([O-])=O)ccc1C(=O)NC(S2)=N[C@@H](c3ccc(F)cc3)C=C2c4ccccc4
**130**	c1cc([N+]([O-])=O)ccc1C(=O)NC(S2)=N[C@@H](c3ccccc3)C=C2c4ccccc4
**131**	c1cc([N+]([O-])=O)ccc1C(=O)NC(S2)=N[C@H](c3ccc(F)cc3)C=C2c4ccccc4
**132**	c1cc([N+]([O-])=O)ccc1C(=O)NC(S2)=N[C@H](c3ccccc3)C=C2c4ccccc4
**133**	c1cc([N+]([O-])=O)ccc1[C@@H]2[NH+]=C(N=C(C)/C2=C(\[O-<br>])OCC)\C=C\c3ccc(F)cc3
**134**	c1cc([N+]([O-])=O)ccc1[C@H]2[NH+]=C(N=C(C)/C2=C(\[O-])OCC)\C=C\c3ccc(F)cc3
**135**	c1ccc(Cl)c([O-])c1/C=C2/C(=O)N(C(=O)S2)Cc3nc(no3)-c4ccccn4
**136**	c1ccc(Cl)cc1COc(ccc2)cc2-c3nc(N)nc(c34)[nH]cn4
**137**	c1ccc(F)cc1CCNC(=O)CN(C(=O)S2)C(=O)\C2=C\c3c([O-])c([N+]([O-])=O)cc(c3)OC
**138**	c1ccc(F)cc1COc(ccc2)cc2-c3nc(N)nc(c34)[nH]cn4
**139**	c1ccc(O)c(Cl)c1/C=C2/C(=O)N(C(=O)S2)Cc3nnc(o3)-c4ccccn4
**140**	c1ccc(O)cc1-c2cn(nn2)Cc3ccc(cc3)-c4ccccc4
**141**	c1ccc(O)cc1-c2cn(nn2)Cc3nnc(o3)-c4cccnc4
**142**	c1ccc(OC)c([O-])c1/C=C2/C(CC)=NN(C2=O)c3c(F)cc(F)cc3
**143**	c1ccc(OC)c([O-])c1/C=C2/C(CC)=NN(C2=O)c3cnccn3
**144**	c1ccc(OC)cc1C(=O)OCc2c(C)n(nn2)-c3nccs3
**145**	c1ccc(OC)cc1C(=O)OCc2cn(nn2)-c(cc3)cc(c34)scn4
**146**	c1ccc(OC)cc1COc(ccc2)cc2-c3nc(N)nc(c34)[nH]cn4
**147**	c1ccc([N+]([O-])=O)cc1C(=O)OCc2c(C)n(nn2)-c3nccs3
**148**	c1ccc([N+]([O-])=O)cc1C(=O)OCc2cn(nn2)-c(cc3)cc(c34)scn4
**149**	c1ccc([N+]([O-])=O)cc1C(=O)OCc2cn(nn2)C[C@@H](O3)COc(c34)cccc4
**150**	c1ccc([N+]([O-])=O)cc1C(=O)OCc2cn(nn2)C[C@H](O3)COc(c34)cccc4
**151**	c1ccc([N+]([O-])=O)cc1C(=O)OCc2cn(nn2)Cc3ccc(cc3)-c4ccccc4
**152**	c1ccc([N+]([O-])=O)cc1C(=O)OCc2cn(nn2)Cc3nc(no3)-c4cccnc4
**153**	c1ccc([N+]([O-])=O)cc1C(=O)OCc2cn(nn2)Cc3nnc(o3)-c4cccnc4
**154**	c1cccc(c12)OC[C@@H](O2)Cn(nn3)cc3-c4cc(O)ccc4

**155**	c1cccc(c12)OC[C@@H](O2)Cn(nn3)cc3-c4ccccn4
**156**	c1cccc(c12)OC[C@H](O2)Cn(nn3)cc3-c4cc(O)ccc4
**157**	c1cccc(c12)OC[C@H](O2)Cn(nn3)cc3-c4ccccn4
**158**	c1cccc(c12)[nH]cc2CCn(nn3)cc3-c4cc(O)ccc4
**159**	c1cccc(c12)[nH]cc2CCn(nn3)cc3-c4ccccn4
**160**	c1cccc(c12)cccc2OCc3cc(ccc3)-c4nc(N)nc(c45)[nH]cn5
**161**	c1cccc(c12)nccc2C(=O)OCc3c(C)n(nn3)-c4nccs4
**162**	c1cccc(c12)nccc2C(=O)OCc3cn(nn3)-c(cc4)cc(c45)scn5
**163**	c1ccccc1/C=C/C(N=C(C)/C2=C(\[O-])OCC)=[NH+][C@@H]2c3cc(ccc3)OCC
**164**	c1ccccc1/C=C/C(N=C(C)/C2=C(\[O-])OCC)=[NH+][C@H]2c3cc(ccc3)OCC
**165**	c1ccccc1C2=C[C@@H](N=C(S2)N)c3ccc(F)cc3
**166**	c1ccccc1C2=C[C@@H](N=C(S2)N)c3ccccc3
**167**	c1ccccc1C2=C[C@H](N=C(S2)N)c3ccc(F)cc3
**168**	c1ccccc1C2=C[C@H](N=C(S2)N)c3ccccc3
**169**	c1ccccc1C(=O)NC(S2)=N[C@@H](c3ccc(F)cc3)C=C2c4ccccc4
**170**	c1ccccc1C(=O)NC(S2)=N[C@@H](c3ccccc3)C=C2c4ccccc4
**171**	c1ccccc1C(=O)NC(S2)=N[C@H](c3ccc(F)cc3)C=C2c4ccccc4
**172**	c1ccccc1C(=O)NC(S2)=N[C@H](c3ccccc3)C=C2c4ccccc4
**173**	c1ccccc1C(=O)c2ccc(cc2)Cn(nn3)cc3-c4cc(O)ccc4
**174**	c1ccccc1C(=O)c2ccc(cc2)Cn(nn3)cc3-c4ccccn4
**175**	c1ccccc1C(=O)c2ccc(cc2)Cn(nn3)cc3COC(=O)c4cc([N+]([O-])=O)ccc4
**176**	c1ccccc1C(=O)c2ccc(cc2)Cn(nn3)cc3COC(=O)c(c4)ccc(c45)OCO5
**177**	c1ccccc1CNC(=O)CN(C(=O)S2)C(=O)\C2=C\c3ccc(cc3)OC
**178**	c1ccccc1COc(c2C)cc(cc2)-c3nc(Cl)nc(c34)[nH]cn4
**179**	c1ccccc1COc(c2C)cc(cc2)-c3nc(N)nc(c34)[nH]cn4
**180**	c1ccccc1COc(ccc2)cc2-c3nc(N)nc(c34)[nH]cn4
**181**	c1ccccc1OC(=O)CN(C(=O)S2)C(=O)\C2=C\c3c([O-])c([N+]([O-])=O)cc(Br)c3
**182**	c1ccccc1OC(=O)CN(C(=O)S2)C(=O)\C2=C\c3cc(O)c(O)cc3
**183**	c1ccccc1OC(=O)CN(C(=O)S2)C(=O)\C2=C\c3cc([O-])c([N+]([O-])=O)cc3
**184**	c1cnccc1C(=O)OCc2c(C)n(nn2)-c3nccs3
**185**	c1ncccc1-c(no2)nc2Cn(nn3)cc3-c4cc(O)ccc4
**186**	n1c[nH]c(c12)nc(N)nc2-c3cc(ccc3)Oc4ccccc4
**187**	n1c[nH]c(c12)nc(N)nc2-c3ccc(I)cc3
**188**	n1c[nH]c(c12)nc(N)nc2-c(cc3C(F)(F)F)cc(c3)OCc4ccc(cc4)OC
**189**	n1ccccc1-c2cn(nn2)Cc3ccc(cc3)-c4ccccc4
**190**	n1ccccc1-c2cn(nn2)Cc3nc(no3)-c4cccnc4
**191**	n1ccccc1-c2cn(nn2)Cc3nnc(o3)-c4cccnc4
**192**	n1ccncc1N(C2=O)N=C(C)\C2=C\c3cc(ccc3)Oc4ccccc4

References

1. Ali, M.M.U.; Roe, S.M.; Vaughan, C.K.; Meyer, P.; Panaretou, B.; Piper, P.W.; Prodromou, C.; Pearl, L.H., Crystal structure of an Hsp90–nucleotide–p23/Sba1 closed chaperone complex. *Nature* **2006**, 440, 1013–1017. https://doi.org/10.1038/nature04716.
2. Lee, C.-C.; Lin, T.-W.; Ko, T.-P.; Wang, A.H.J., The Hexameric Structures of Human Heat Shock Protein 90. *PLoS One* **2011**, *6*, e19961. https://doi.org/10.1371/journal.pone.0019961.
3. Terracciano, S.; Chini, M.G.; Vaccaro, M.C.; Strocchia, M.; Foglia, A.; Vassallo, A.; Saturnino, C.; Riccio, R.; Bifulco, G.; Bruno, I., Correction: Identification of the key structural elements of a dihydropyrimidinone core driving toward more potent Hsp90 C-terminal inhibitors. *Chem. Commun. (Camb.)* **2016**, *52*, 13515. https://doi.org/10.1039/c6cc90506d.
4. Vassallo, A.; Vaccaro, M.C.; De Tommasi, N.; Dal Piaz, F.; Leone, A., Identification of the Plant Compound Geraniin as a Novel Hsp90 Inhibitor. *PLoS One* **2013**, *8*, e74266. https://doi.org/10.1371/journal.pone.0074266.
5. Dal Piaz, F.; Vassallo, A.; Temraz, A.; Cotugno, R.; Belisario, M.A.; Bifulco, G.; Chini, M.G.; Pisano, C.; De Tommasi, N.; Braca, A., A chemical-biological study reveals C9-type iridoids as novel heat shock protein 90 (Hsp90) inhibitors. *J. Med. Chem.* **2013**, *56*, 1583–95. https://doi.org/10.1021/jm301398y.
6. Strocchia, M.; Terracciano, S.; Chini, M.G.; Vassallo, A.; Vaccaro, M.C.; Dal Piaz, F.; Leone, A.; Riccio, R.; Bruno, I.; Bifulco, G., Targeting the Hsp90 C-terminal domain by the chemically accessible dihydropyrimidinone scaffold. *Chem. Commun.* **2015**, *51*, 3850–3853. https://doi.org/10.1039/c4cc10074c.
7. Chini, M.G.; Malafronte, N.; Vaccaro, M.C.; Gualtieri, M.J.; Vassallo, A.; Vasaturo, M.; Castellano, S.; Milite, C.; Leone, A.; Bifulco, G.; De Tommasi, N.; Dal Piaz, F., Identification of Limonol Derivatives as Heat Shock Protein 90 (Hsp90) Inhibitors through a Multidisciplinary Approach. *Chem. Eur. J.* **2016**, *22*, 13236–13250. https://doi.org/10.1002/chem.201602242.
8. Terracciano, S.; Russo, A.; Chini, M.G.; Vaccaro, M.C.; Potenza, M.; Vassallo, A.; Riccio, R.; Bifulco, G.; Bruno, I., Discovery of new molecular entities able to strongly interfere with Hsp90 C-terminal domain. *Sci. Rep.* **2018**, *8*. https://doi.org/10.1038/s41598-017-14902-y.
9. D'Ambola, M.; Fiengo, L.; Chini, M.G.; Cotugno, R.; Bader, A.; Bifulco, G.; Braca, A.; De Tommasi, N.; Dal Piaz, F., Fusicoccane Diterpenes from Hypoestes forsskaolii as Heat Shock Protein 90 (Hsp90) Modulators. *J. Nat. Prod.* **2019**, *82*, 539–549. https://doi.org/10.1021/acs.jnatprod.8b00924.
10. Teracciano, S.; Chini, M.G.; Vaccaro, M.C.; Strocchia, M.; Foglia, A.; Vassallo, A.; Saturnino, C.; Riccio, R.; Bifulco, G.; Bruno, I., Identification of the key structural elements of a dihydropyrimidinone core driving toward more potent Hsp90 C-terminal inhibitors. *Chem. Commun.* **2016**, *52*, 12857–12860. https://doi.org/10.1039/c6cc06379a.
11. Madhavi Sastry, G.; Adzhigirey, M.; Day, T.; Annabhimoju, R.; Sherman, W., Protein and ligand preparation: parameters, protocols, and influence on virtual screening enrichments. *J. Comput.-Aided Mol. Des.* **2013**, *27*, 221–234. https://doi.org/10.1007/s10822-013-9644-8.
12. *Protein Preparation Wizard; Epik,* New York, NY, USA, 2017.
13. Friesner, R.A.; Murphy, R.B.; Repasky, M.P.; Frye, L.L.; Greenwood, J.R.; Halgren, T.A.; Sanschagrin, P.C.; Mainz, D.T., Extra precision glide: Docking and scoring incorporating a model of hydrophobic enclosure for protein−ligand complexes. *J. Med. Chem.* **2006**, *49*, 6177–6196. https://doi.org/10.1021/jm051256o.
14. Friesner, R.A.; Banks, J.L.; Murphy, R.B.; Halgren, T.A.; Klicic, J.J.; Mainz, D.T.; Repasky, M.P.; Knoll, E.H.; Shelley, M.; Perry, J.K., Glide: a new approach for rapid, accurate docking and scoring. 1. Method and assessment of docking accuracy. *J. Med. Chem.* **2004**, *47*, 1739–1749. https://doi.org/10.1021/jm0306430.
15. Halgren, T.A.; Murphy, R.B.; Friesner, R.A.; Beard, H.S.; Frye, L.L.; Pollard, W.T.; Banks, J.L., Glide: a new approach for rapid, accurate docking and scoring. 2. Enrichment factors in database screening. *J. Med. Chem.* **2004**, *47*, 1750–1759. https://doi.org/10.1021/jm030644s.
16. *Glide*; New York, NY, USA, 2017.
